# Practical challenges in oral immunotherapy resolved through patient-centered care

**DOI:** 10.1186/s13223-021-00533-6

**Published:** 2021-03-18

**Authors:** François Graham, Douglas P. Mack, Philippe Bégin

**Affiliations:** 1 Allergy and Immunology, Centre Hospitalier de L’Université de Montréal, Hôpital Notre-Dame, Montreal, QC Canada; 2 Allergy and Immunology, Centre Hospitalier Universitaire Sainte-Justine, 3175 Chemin de la Cote Sainte-Catherine, Montréal, QC H3T1C5 Canada; 3 Department of Pediatrics, McMaster University, Hamilton, ON Canada

**Keywords:** Oral immunotherapy, Food allergy, Peanut allergy, Multi-food oral immunotherapy, Oral food challenge, Shared decision-making, Patient-centered care

## Abstract

Oral immunotherapy (OIT) is now widely recognized as a valid option for the management of IgE-mediated food allergies. However, in real-life practice, OIT can lead to a variety of unique situations where the best course of action is undetermined. In patient-centered care, individual patient preferences, needs and values, should guide all clinical decisions. This can be achieved by using shared-decision making and treatment customization to navigate areas of uncertainty in a way that is responsive to patient’s needs and preferences. However, in the context of OIT, lack of awareness of potential protocol adaptability or alternatives can become a barrier to treatment personalization. The purpose of this article is to review the theoretical bases of patient-centered care and shared decision-making and their practical implication for the patient-centered delivery of OIT. Clinical cases highlighting common challenges in real-life OIT practice are presented along with a discussion of potential personalized management options to be considered. While the practice of OIT is bound to evolve as additional scientific and experiential knowledge is gained, it should always remain rooted in the general principles of patient-centered care.

## Introduction

Oral immunotherapy (OIT) is recognized as a reasonable alternative to strict avoidance for the management of IgE-mediated food allergies. OIT protocols generally consist of daily ingestion of the offending food allergen (food dosing), starting below a patient’s eliciting dose (i.e. the smallest quantity of allergen that would elicit an allergic reaction), and increasing the dose over time with the objective of increasing clinical tolerance to that food.

Recent Canadian guidelines have made clear recommendations concerning the initiation of OIT for the treatment of patients with IgE-mediated food allergy [1], based on evidence and a diversity of ethical imperatives. The guidelines specifically emphasized the importance of developing personalized and patient-centered approaches to food allergy management rather than following a “one-size-fits-all” approach, in order to maximise its impact and relevance for patients and families. This said, the Canadian guidelines also recognized that the personalized care in OIT represents additional challenge in terms of implementation and training. There is also confusion as to the actual meaning of shared decision-making (SDM) and patient-centered care (PCC).

The purpose of this review is to present practical aspects of oral immunotherapy treatment personalization in real-life aimed at clinicians interested in developing an OIT clinical offer rooted in the principles of PCC. In the first part, the article clarifies the meaning of PCC and SDM. In the second part, the article introduces cases depicting common clinical challenges seen in OIT practice where there is no clear single best approach. These are meant to serve as a practicum illustrating the creation of choice awareness in real life rather than to provide a “right answer”. In fact, the objective is not to provide definitive answers to each case but rather to provide clinicians with examples of adaptive thinking and SDM that will help them determine the “right answer” for each individual patient, which should be the standard approach for the optimal delivery of patient-centered OIT.

## Patient-centered care

The concept of PCC was introduced in 1988 with the goal of shifting the focus of healthcare “from diagnosis and management of diseases to the needs and desires of patients and their families” [2]. It has since been established as one of the 6 domains of the healthcare quality by the Institute of Medicine (IoM) [3], along with being safe, effective, timely, efficient and equitable. The IoM defines PCC as “providing care that is respectful of, and responsive to, individual patient preferences, needs and values, and ensuring that patient values guide all clinical decisions” [3].

**Fig. 1:**
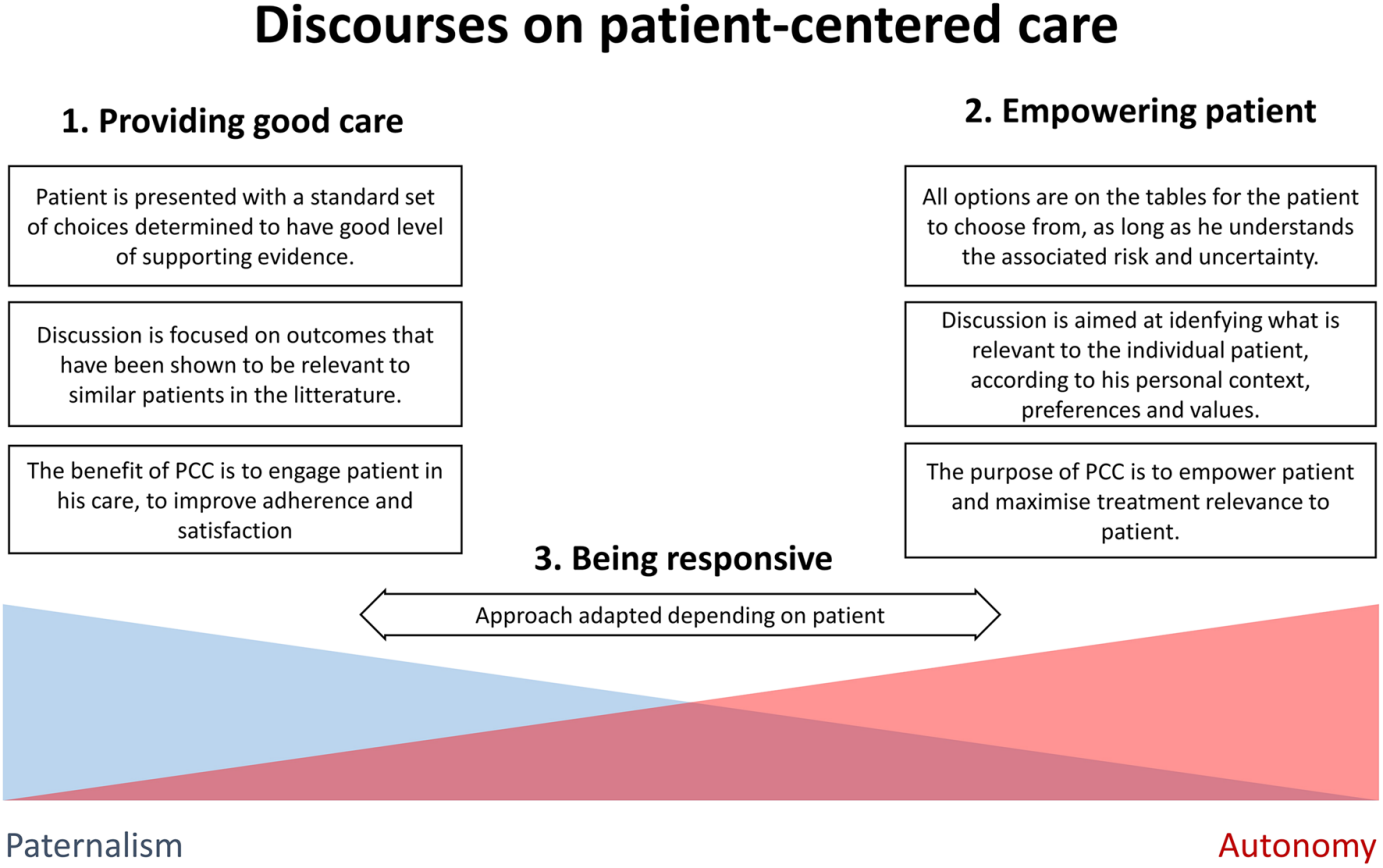
Discourses on patient-centered care. The diagram describes three distinct discourses on patient-centered care found in the medical literature in an analysis performed in 2015 [4]

The term PCC is now widely used in the medical literature but with various meanings. In 2015, an analysis of the literature identified three distinct discourses on PCC (Fig. 1) [4]. The first discourse is developed around the idea of providing good care and engaging patients through holistic approaches to improve adherence and satisfaction with treatment plan. While this is positive, the discourse remains anchored in a relatively paternalistic vision of medicine where doctors ultimately decide what is in the best interest of their patients, and the actual impact of patient preferences on decisions is unclear.

The second discourse presents PCC as providing patients with the means to make decisions for themselves, according to their own perspectives and values. This is a purist vision of PCC, with patient empowerment as an important underlying theme. One criticism has been that, even with proper information, patients may not always know what the best option for them is (e.g. opioid addiction) or may simply prefer allowing the doctor to decide.

The third discourse on PCC presents it as “being responsive” to the patient’s individual needs, adjusting the extent to which he participates to healthcare decisions according to context and his desire. The “responsive” vision of PCC can allow the patient to make certain decisions but leave others to the clinician. Most importantly, if a decision is made by the doctor, it must reflect the individual patient’s preferences, experiences and values, implying that those still need to have been thoroughly explored with the patient beforehand through shared-decision making approaches.

## Shared-decision making

SDM describes the process through which patients are made to play an active role in the management of their health [5]. The key elements of SDM are the active involvement of patient in the treatment plan, sharing of information, expressing preferences, and mutual agreement of the course of treatment [6]. SDM should not be mistaken for a more extensive informed consent. With informed consent, the patient’s “choice” is limited to accepting or rejecting a proposed path that has been identified as most relevant for them [7, 8]. In contrast, SDM is not limited to a finite time period, but rather involves a continuous conversation between clinician and patient in which the patient learns about the disease and treatment options and the clinician learns about the patient’s values and preferences. In OIT, patients and clinicians jointly begin this process at the initial consultation, develop it further during subsequent interactions including the counselling/consent visit and revisit this process as challenges arise during the treatment regimen. Through these exchanges they come to a mutual understanding of the potential options that would best fit with what matters to the patient allowing the patient and clinician to design and manage a customized and comprehensive care plan [9]. Decision aids can be useful tools to involve patients and share information on OIT, although they should not be solely relied upon to assess patient preferences and personalize treatment plan [10–12].

**Fig. 2:**
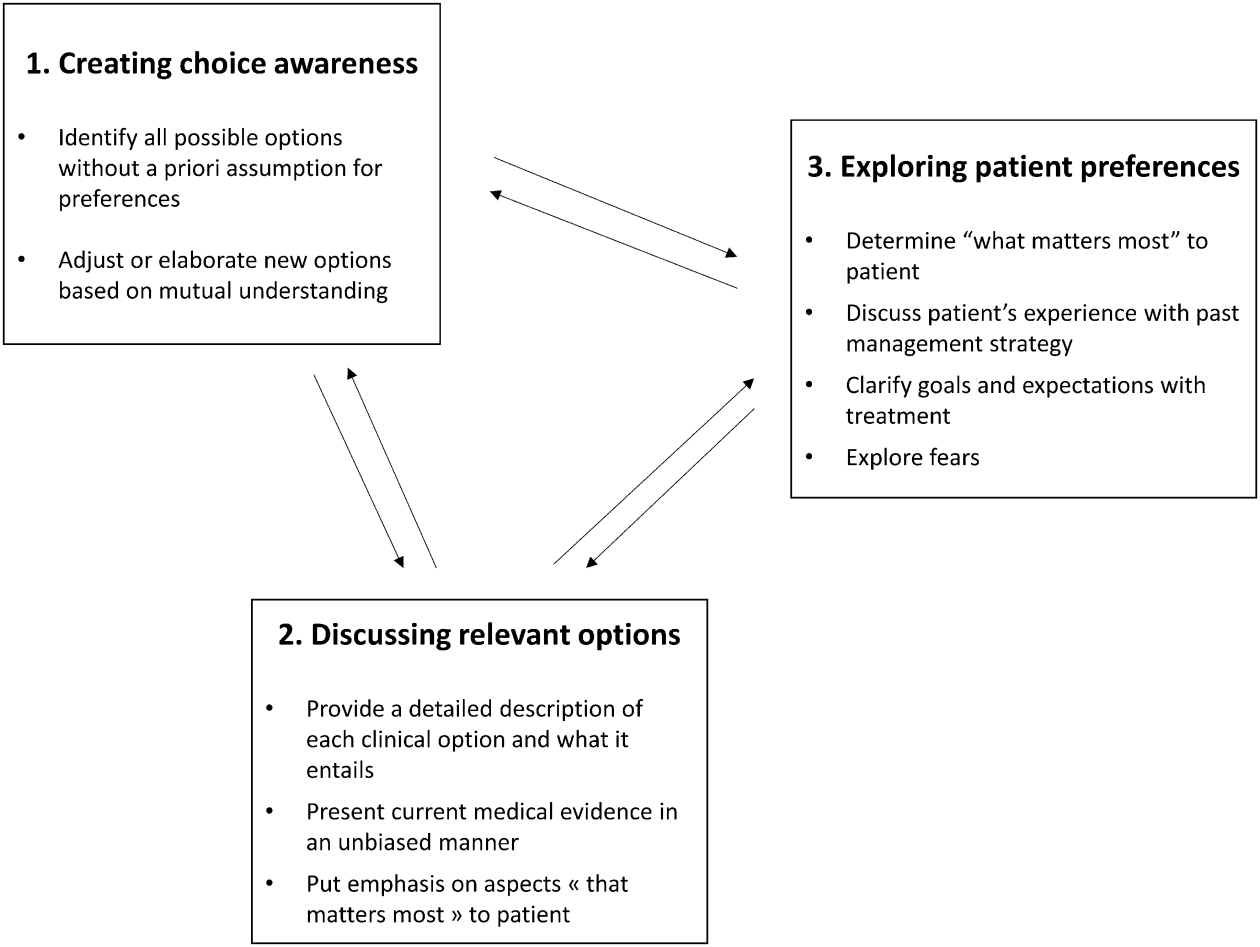
Shared-decision making process. Diagram depicts the 3 steps of shared-decision making

Most models for SDM include at least 3 steps or components which are: (1) creating choice awareness, (2) discussing relevant options and (3) discussing patient preferences [13–16] (Fig. 2). These should be integrated in a fluid conversation allowing for back and forth between the steps as discussions generate new options to consider.

Creating choice awareness is a critical component of SDM. Patients cannot be proactive in determining care direction unless they know there is more than one sensible way to address their condition and therefore a decision to be made [13]. In fact, lack of choice awareness has been identified as a key limitation in the patient-centeredness of informed consent [8, 17]. Creating choice awareness is most relevant when there is uncertainty surrounding a “single best” treatment option because patient preferences should then weigh even more in the final decision [2, 5, 18]. In the context of food allergy, the various ways to approach avoidance and OIT generate a very large set of possible choices for patients and one can hardly suggest that there is a unanimous consensus on a unique approach that will best answer the needs of all patients. This makes SDM approaches all the more relevant to practice in this field [19].

In order to offer choices to patient, clinicians must be aware of the possible options themselves and randomized clinical trials provide limited insight in terms of protocol adaptability for clinicians new to OIT. The second part of this review presents practical examples of alternative approaches that can be considered in the real-life practice of OIT in order to foster choice awareness as part of a SDM process. The first case is used to illustrate the different components of the SDM process whereas the following cases focus on the creation of choice awareness.

## Case discussions

### Case 1

A 2-year-old Caucasian male with a history of urticaria after ingestion of peanut butter at 1 year of age. Skin prick testing (SPT) with peanut extract reveals a 4 mm wheal diameter. Peanut and Ara h 2 serum-specific IgE (ssIgE) are respectively 1.0 and 0.4 kU/L. Parents refuse an oral food challenge (OFC) but are interested in OIT.

Barriers to OFCs are well recognized with both physicians and patients reporting hesitation. Recent studies have demonstrated low rates of OFC performance amongst allergists [20]. Clinicians report concern with potential reactions, reimbursement and clinic space or support staff as reasons for limiting OFCs [21–23]. Parents cite anxiety and lack of information as reasons for reluctance [21].

In principle, patients should not perform OIT for foods they are not allergic to and accurate diagnostic approaches should be implemented whenever the diagnosis is in question [1, 24]. These approaches may include SPT and ssIgE but they have limited predictive value and the gold-standard remains OFCs [25]. Development of novel approaches with improved diagnostic accuracy (e.g. basophil activation tests, conjunctival provocation tests) could potentially help to guide decision making for OIT in the future [26–30].

In this case, the parent’s diverging view on the question suggests OFC being the single “best approach” is not self-evident and indicates the need for SDM. Before proceeding, the clinician should make sure to validate the parents’ perspective in a non-judgmental manner to engage them in the collaborative process, especially if he has just expressed a very strong opinion in favor of the “standard” approach. Recognizing that patients and physicians perceive risk differently based on their clinical knowledge, general tolerance of risk and past experience can help the clinician demonstrate openness.

### Creating choice awareness

The clinician should not limit the choices to “standard” options but rather present parents with all reasonable options to be openly discussed and seriously considered. In the ensuing conversation, the clinician will get to explain why they are not considered standard-of-care as a general rule, and the patients will get to express how this option resonates with his individual context and needs. In this specific case, potential choices to consider should include at least: (A) not performing the OFC and continuing with strict avoidance; (B) performing the OFC and deciding what to do next based on its outcome; and (C) performing OIT without prior OFC.

### Describing the options

Each possible option should be examined individually, with the clinician providing an unbiased assessment of pros and cons based on available knowledge and taking into account data on patient-reported outcome when available.

#### Continue avoidance

Even if continuing avoidance does not constitute a change from the current management strategy, it should still be discussed as an option rather than simply treated as default (i.e. patient is opting for status quo). Reviewing previous experiences in terms of severity and frequency of reactions and the impact of avoidance in terms of both limitations and feeling of safety can help provide valuable insight into the patient and family’s motivations and expectations with regards to the other options. Health care providers should use caution when discussing statistics since the concept of uncertainty generalizability can be hard to grasp for some patients (and even clinicians). Patients with low numeracy can have inflated impression of what a specific number means and perceive it as a certainty [31, 32]. Switching the framing (from positive to negative) and the format (from percentage to frequency) can help ensure the outcomes are understood [33]:

“Studies show that 22% of peanut allergic children will have resolved their allergy by 4 years of age [34]. This means that 4 out of 5 will still be allergic by that age.”

Also, options should be presented in light of specific patient context. For example, in the context of a family refusing OFC, a watchful waiting approach may be complicated by the fact that the family may keep refusing OFC in the future and potentially never introduce the food.

#### Performing the OFC

From a medical standpoint, the main advantage of an OFC in the current case is that it may reveal that the patient is not allergic, and therefore does not require OIT. Alternatively, if the patient was to be found allergic, an OFC would still provide valuable information about reactivity threshold, which can be useful for personalizing the OIT schedule or determining the extent of avoidance required. It may also contribute to a more accurate recognition of symptoms that require immediate treatment and thus improve understanding and management of future reactions to OIT doses at home. Finally, food OFCs have a beneficial effect on quality of life by clarifying the severity of the food allergy and reducing anxiety when confronted with accidental reactions [35–38]. In fact, the parents’ reluctance to perform OFC in this case suggests they would most likely benefit from undergoing one.

However, OFCs do carry the risk of allergic reaction and while typically these reactions are mild, severe and even fatal reactions have occurred [39]. The risk of reactions has also been noted to be higher when OFC was performed with the specific purpose of confirming the allergy prior to OIT [40]. Parental fear of this risk remains a barrier to successful completion of OFC and it is important to recognize and validate the parents’ and patient’s concern of potential reaction.

#### Proceeding directly to OIT

Conversely, the main risk of proceeding to OIT without first doing the OFC is that if the patient is not allergic, all the OIT visits and daily doses are unnecessary. The opportunity cost associated with unnecessary OIT can be substantial [1], especially if the patient is left on therapy in the long term without ever rechallenging the diagnosis or testing for sustained tolerance. The burden is even greater for patients with food aversion.

On the other hand, assuming that the patient was indeed not allergic, proceeding directly to OIT does not present medical risk and will in the end allow the introduction of the food with the same benefit on quality of life as would the OFC, albeit with a greater time and effort investment [41, 42]. Rather than discarding this time investment as a waste, the clinician should simply describe it objectively in order to let the patient and family decide for themselves whether it is worth it.

Finally, parents afraid of OFC reactions but interested in OIT should be warned of the real risk of reacting to doses at home during treatment. As in all patients undergoing OIT, they should have a personalized food allergy action plan to help them recognize and manage reactions occurring at home during therapy and undergo training for epinephrine autoinjector utilization [1]”.

### Exploring preferences

Once all the options have been explained to the family, the discussion should be focus on exploring and respecting “what matters most” to the patient and his family as individuals.

In this example, some clinicians may feel it is illogical for the parents to refuse an OFC for fear of reaction while accepting reactions from OIT. The fact that the patient’s position does not make sense to the clinician should act as a cautionary warning that the clinician may have failed to grasp the patient’s perspective. Many patients find dosing reactions to be acceptable because they are perceived as unavoidable side effects from a necessary treatment [43], whereas the benefit from OFC may be perceived as more theoretical and insufficient to justify the risk of severe reaction.

While the health care provider should be careful not to push for a mandatory OFC when parents have clearly indicated it is not for them, it is important to understand the reason for the refusal. In this example, the clinician may realize that the parents are not completely averse to the idea of an OFC but are worried about the need to go “all the way” until there is a reaction. They may be willing to consider performing a challenge up to a maximum amount that, if passed, would enable the use of less intensive high-threshold OIT/food introduction protocols (see Case 2). Patient’s response to such personalized approaches is often that they “did not think this was allowed” (i.e. creating choice awareness). While this approach would not completely rule out the possibility that the patient may not be allergic, it would allow food introduction and it could be presented as first step preparing them for an eventual subsequent OFC that would “go all the way”.

#### Mitigating the risk of bias

All patients in whom OIT is being considered should undergo a detailed discussion of risk and benefits of treatment, potential outcomes, and clinicians should address patient and caregivers’ concerns and expectations. Even with the most patient-centric decision-making approaches, patients will still depend on physician’s opinion to guide their decision and physicians must thus be mindful of their own biases when presenting options. Biases are unconscious thinking patterns that influence our perception, and therefore our presentation of the clinical options in a more or less favorable manner. Cognitive bias result from faulty reasoning whereas affective bias result from emotional factors. Various types of biases have been described in the context of decision-making and clinical reasoning, some of which are listed in Box 1.

Shared decision-making is particularly vulnerable to cognitive and affective biases because it is used in contexts where there is high level of uncertainty and largely depends on human judgment and cognitive flexibility [44]. On one hand, opening the door to performing OIT without confirming diagnosis gives more options to patients. However, it can give allergists an excuse to avoid the inconvenience of an OFC and perform easy OIT in low-risk patients. In fact, some allergy clinics appear to have built a “highly successful” business model of unnecessarily treating individuals that are not actually allergic. It should be made very clear that the latter is medical fraud, which has nothing to do with cognitive biases or SDM. This said, decision-making behaviors are directly affected by anticipated cognitive demand and when given the option, even honest individuals will tend to instinctively favor the option that requires the least mental effort (law of least effort bias) [45].

Recognizing biases requires introspection and they are often easier to detect in others than for ourselves (blindspot bias) [46]. When recognized, bias can be partly compensated by attempting to suppress the intuitive response and rebalancing the discourse (debiasing) [47]. Another way to mitigate the risk of bias is to disclose them in a transparent manner. Paradoxically, studies have shown that physician disclosure of bias leads to greater patient agreement for the proposed intervention, most likely due to the increased trust [48].

Decision aids and checklists can also be useful tools to help mitigate the risk of bias.

One study described the use of a checklist based structure to ensure that all major aspects were discussed in a balanced manner when consenting patients for OIT [12]. In cancer clinical trials, decision aids have been found to reduce patients’ decision regrets [49]. Ideally, details of discussions and the rationale for the final decision should be documented in medical charts, where they can be periodically verified with internal audits. Periodic chart review for quality assessment can also serve to identify inappropriate drifts in clinical practice.

Box 1: Selected cognitive biases that can influence physician inclination towards OIT *Anchoring effect:* The initial experience with OIT will “anchor” the physician’s opinion and will taint the perception of all future decisions (either positively or negatively).

*Blind spot bias:* Tendency to detect bias in others but not in oneself.

*Confirmation bias:* Physician tends to look for evidence supporting their previous position and ignores evidence that does not support it.

*Curse of knowledge:* A well-informed physician that has difficulty understanding the problem from the perspective of less-informed patients.

*Dunning–Kruger effect:* The tendency of unskilled physicians to overestimate their abilities and of skilled physicians to underestimate their abilities.

*Experience limitation bias:* Physician limits options to those that he has previously experienced.

*Law of least effort bias:* A tendency to choose the option that will require the least mental effort.

*Plan continuation bias:* A tendency to persevere with the original plan even though the situation has changed or is not what was expected.

*Post-purchase rationalization (sunk-cost):* A tendency to persist with an ineffective intervention because of the previously invested time and resources.

*Pro-innovation bias:* A physician that tends to be overly optimist about new treatments or technologies and that fails to see their limitations and weaknesses.

*Projection bias:* A physician with food allergy that assumes food allergic patients share the same reality.

*Status quo bias (inertia, conservatism):* A physician that would like practice to remain relatively the same, without major changes.

*Zero-risk bias:* Tendency to prefer reducing a small risk to zero over a more significant reduction of a large risk to a small one.

### Case 2

A 3-year-old presents with mild local reaction at 1 peanut and systemic urticaria at 4 peanuts on his OFC.

#### Source of uncertainty

What is the best approach to patients with a high reaction threshold on OFC?

#### Options to consider with family

(A) Continue with strict avoidance, (B) perform conventional OIT, (C) perform high-threshold OIT, (D) continue strict avoidance of peanuts but allow foods with precautionary allergen labeling.

#### Points to explore/discuss

Many food-allergic patients have high tolerance thresholds at baseline [50], which may sometimes be even higher than the typical target dose for OIT. High threshold for peanut has been defined as a mild reaction to a cumulative eliciting dose of more than 100 mg peanut on OFC [50]. One option for these patients is to initiate OIT directly at that high dose [50, 51]. This “high threshold OIT” can ensure the high threshold is maintained and possibly raised further. In younger children, it could potentially help promote the resolution of the allergy over time. The peanut-sensitized patient subgroup in the LEAP study provides some evidence to support this concept [52].

Compared to regular OIT, high threshold OIT is easier to implement in practice since it generally carries a low risk of reactions and may not involve the preparation of very small individual food doses or regular up-dosing visits [50, 53]. If up-dosing visits are needed, these can occur less frequently (e.g. at 2 or 3 months intervals). In addition, maintenance dose can be achieved faster than conventional OIT due to higher initial starting dose. Maintenance dose target should be discussed and based on the preferences of patients and their caregivers (i.e. desire to introduce large quantities of foods or only protection against accidental reactions). High maintenance doses are generally not pursued unless specifically asked by patients (i.e. desire to eat peanut butter on a daily basis), as studies have shown that low dose OIT (ex: 1 peanut a day) can confer protection to much higher doses in the long run [54, 55]. Furthermore, selecting a lower dose may improve adherence.

Another option in patients with high reactivity thresholds is to relax avoidance to allow traces or small amounts of the food to be eaten without performing OIT [56]. Patients should be informed that the reactivity threshold may decrease over time and it is unclear how irregular exposure will affect the threshold in the long term.

### Case 3

Parents of a 12-month-old girl with raw and baked egg allergy are asking whether they should consider OIT right away or wait and see if the allergy resolves on its own.

#### Source of uncertainty

What is the optimal management of very young children with food allergy?

#### Options to consider with family

(A) Continue with strict avoidance (“watch and wait”), (B) perform OIT.

#### Points to explore/discuss

The frequent resolution of egg and milk allergy in young children poses a specific dilemma when considering OIT. Where some guidelines suggest waiting to see if the allergy resolves spontaneously before performing OIT [24], the high rate of resolution must be balanced against the fact that these ubiquitous allergens have been associated with the highest impact on quality of life, when persistent [57]. Data from large uncontrolled cohort of early intervention in toddlers desensitized for milk allergy suggest it is very well tolerated in this age group [58]. There is uncertainty to which extent the very high tolerance rate (98.1%) in these patients in a median of 106 days is due to the early intervention or natural evolution but it at least indicates a very low burden of therapy in this age group. In this context, parents may not want to risk missing the opportunity of the favorable conditions generated by low IgE in early age to intervene before it risks becoming more challenging to desensitize [59]. Furthermore, despite a high probability of spontaneous resolution by adolescence, many parents may feel that the impact of avoidance on their quality of life persisting through pre-school and school years is sufficient in itself to justify intervention [60, 61]. Ultimately, the key is to create choice awareness around the possibility early desensitization and to base the final decision on parents’ preferences and tolerance to risk.

### Case 4

Parents of a 2-year-old female of Indian descent with multiple confirmed food allergies including peanuts, milk, soy, legumes and tree nuts are interested in OIT.

Over one-third of food-allergic patients have multiple food allergies [62, 63] and managing these poly-allergic patients with OIT poses some unique challenges, including (a) how to determine which food to include/prioritize in the treatment plan and (b) how to plan the multiple desensitizations.

#### Source of uncertainty

What are the best criteria to prioritize foods for OIT in patients with multiple food allergies?

#### Options to consider with family

Base choice on (A) easiest foods to desensitize, (B) on most severe food allergies, (C) on allergens most likely to cause accidental reactions, or (D) on foods with the highest impact on day-to-day life.

#### Points to explore/discuss

While some families may have a clear idea on which food to prioritize from the outset, most will seek guidance or validation from their allergist. Developing a preference-sensitive plan with families through SDM is crucial to ensure the benefits from treatment are maximized. After presenting the four options mentioned above, the clinician should ask the patient to reflect on what motivated them to seek OIT in the first place and what are their expectations from treatment. In the example above, the presence of tree nuts such as cashew and pistachio in the family’s diet may make these foods a priority over milk or soy [64]. In older patients, treatment objectives are often shaped by past experiences of reaction, social limitations or missed opportunities attributed to the allergies [65]. For example, young adults will often prioritize allergens that prevent them from travelling or socializing.

The relevance of allergy test results for prioritizing allergens again depends on patient’s personal goals. Some patients may feel more comfortable starting with “milder allergens” to test the water before moving on with other foods if well tolerated. Conversely, others may find starting with their most “severe” allergens will help them achieve their objectives faster and improve their quality of life. In the end, the main criteria for prioritizing food allergens should be their relevance for the patient or family.

#### Source of uncertainty

What is the best approach to desensitize multiple foods?

#### Options to consider with family

(A) Include many foods in a multi-food OIT protocol, or (B) approach each food individually, in sequence.

#### Points to explore/discuss

Performing OIT simultaneously for multiple foods has the benefit of reducing the number of cycles translating into reduced amount of time and personal/healthcare cost and could capitalize on young age and lower IgE, or current access to biologics [1, 66, 67]. The approach is usually to treat the all food as a single entity with a constant stoichiometric ratio, so they will progress together. Managing OIT to multiple foods simultaneously can however increase the burden of treatment, especially when the patient develops food aversions. It also adds a layer of complexity to the management of side-effects, when attempting to identify the culprit food.

The sequential approach may be simpler to implement and manage for some clinicians and patients. It also allows to provide a learning experience for the family with a less severe allergen (i.e. low hanging fruit) that can help determine whether they wish to proceed with subsequent foods.

### Case 5

Parents of a 3-year-old boy are asking if desensitizing his wheat allergy will improve his allergy to barley and rye.

#### Source of uncertainty

What is the best approach for OIT to cross-reactive foods?

#### Options to consider with family

(A) Desensitize both foods, (B) desensitize the dominant allergen and check for bystander effect, (C) investigate cross-reactivity in vitro.

#### Points to explore/discuss

In patients with multiple food allergies, a specific consideration should be given to allergen cross-reactivity. “Cross-reactivity” refers to clinical reactivity to foods with similar protein structures, whereas the term “co-sensitization” refers to multiple, unrelated sensitizations to several structurally unrelated allergen groups [68, 69]. In OIT, “cross-desensitization” is when desensitization with one allergen modulates clinical reactivity to a related cross-reactive allergen that is not included in the OIT mix.

Desensitization to cashew will generally provide at least partial protection for pistachio [70–72] and the same phenomenon is seen with walnut OIT for pecan [70, 73]. The opposite (i.e. pistachio OIT for cashew desensitization, and pecan OIT for walnut desensitization) has not been specifically studied in clinical trials but clinical practice does not suggest any difference.

For some foods such as cereals and legumes, the extent of cross-desensitization is unclear. One option can be to adopt a sequential approach, starting with the main allergen from the group and performing OFC to related allergens once maintenance is achieved. The risk with this approach is that if there is no bystander desensitization, the patient will need to undergo subsequent OIT cycle for these foods. The other option is to include all potentially cross-reactive foods simultaneously in the treatment plan but this approach could needlessly increase the amount of food taken daily and affect treatment burden and long- term adherence.

An alternative option could be to investigate cross-reactivity in vitro (ex: IgE inhibition assays) [74–79]. However, such assays are not widely available and have not been studied in the specific context of OIT. Overall, further clinical data is required to better understand cross-desensitization in the context of multi-food OIT.

### Case 6

An 18-month-old currently desensitized for egg, mustard and cashew develops a new allergy to hazelnut.

#### Source of uncertainty

What is the best management of new food allergies arising during OIT?

#### Options to consider with family

(A) Complete the first OIT and then address the new food allergy, (B) modify the current OIT mix to integrate the new allergen.

#### Points to explore/discuss

Desensitizing at an early age comes with the increased risk of new food allergies developing after an OIT program has been initiated. It is good practice to ensure all major allergens have been introduced before beginning OIT, but new allergies can still arise in previously tolerated foods. When this happens, the options are to either add the new allergen in the treatment plan, or to treat it subsequently. Again, the decision should rest with parents. If the decision is to include the allergen, one approach is to keep the food mix at its current daily dose while OIT is initiated with the new allergen until it has caught up with the other allergens, and then resume dosing with the new mix including the new allergen.

### Case 7

A 7-year-old boy with multiple severe food allergies experiences recurrent anaphylactic reactions despite appropriate avoidance strategies. He lives in a northern rural area where allergy clinical offer is limited to the patient’s general pediatrician who does have experience with food challenges. Parents are very interested in OIT.

#### Source of uncertainty

What is the best management of OIT cases in remote areas with limited access to specialized allergy care?

#### Options to consider with family

(A) Continue with strict avoidance, (B) offer conventional OIT at institution with OIT expertise and have the patient travel for treatment, (C) support pediatrician in offering OIT in patient’s region, (D) perform omalizumab-enabled accelerated OIT protocol.

#### Points to explore/discuss

In this case, even if the family has a clear preference for OIT, this option is limited by the availability of a specialized clinical offer in their region, which poses a specific challenge in terms of equity in access to care, which is another of the 6 domains of healthcare quality. Even if traveling costs are assumed by the province following the Canadian Health Act [80], the burden of flying or driving to an OIT specialized center may remain prohibitive for many families.

Supporting the patient’s pediatrician to initiate conventional OIT is an avenue that could be explored by offering OIT training either by telemedicine or as a clinical rotation in a specialized center. Building this type of collaborative approach poses specific challenges but the new structure and accelerated knowledge transfer could be seen as an investment that would ultimately benefit all patients in this area. Before initiating such endeavours, one important aspect to consider is whether the pediatrician has adequate motivation, training, infrastructure and multidisciplinary team support to perform OIT locally. It may be safer to first pilot such a collaborative approach with patients presenting prognostic factors for an “easier” desensitization [81–84] in order to avoid exhausting local resources.

Another option to explore in challenging cases would be omalizumab-enabled OIT (OEAOIT) [1]. Previous studies have shown that omalizumab can increase the safety of desensitization, allowing for an accelerated up-dosing schedule translating in reduced number of up-dosing visits [71, 85, 86]. During the pre-treatment phase, which usually lasts for 2 months, the injections can be administered at home or with the pediatrician [87] prior to the in-person visit with the allergist for OIT. The main downside is the cost of medication, which in this case would be offset by the cost of traveling. Buy-in from the local pediatrician is still required but this approach likely has greater feasibility and acceptability than performing regular multi-food OIT at distance. Ultimately, the local care provider needs to be involved in the shared decision-making process.

### Case 8

An 8-year girl has just started peanut OIT and her family refuses to come in to the clinic during the COVID-19 pandemic. They ask about the possibility of up-dosing at home?

#### Source of uncertainty

Is up-dosing at home feasible and reasonable for this patient?

#### Options to consider with family

(A) Refuse up-dosing at home and remain on current dose until the end of the pandemic, or (B) establish a personalized home-based up-dosing plan with family.

#### Points to explore/discuss

The first step in this case would be to explore patient fears toward coming in clinic and provide up-to-date information about community transmission rate and processes in place to mitigate the risk of infection in place. It should be made clear from the start that the current standard of care in OIT is for up-dosing to occur in clinic [88], and that most of the protocols in the literature were performed that way. Outside of safety considerations, regular updosings at the clinic are beneficial as they enable regular monitoring by the treating team and are an opportunity to answer questions and address educational needs. Moreover, if a reaction occurs at home following up-dosing, they must be prepared to go the emergency department. In the context of the pandemic, it can be argued that the safest option would be to hold current build-up dose for an extended period until able to return for up-dosing at the allergy clinic [89].

That being said, the clinician should acknowledge that experience up to now shows that, apart for the initial escalation visit, the rate of reactions does not appear significantly higher with up-dosing in clinic compared to home dosing and that the majority of severe reactions from OIT is already expected to occur at home [90, 91]. A retrospective review of 1037 patients undergoing OIT in one specialized center suggests asthma, pre-OIT reaction severity, lower tolerated dose, and epinephrine-treated reactions during the initial clinic up-dosing could serve as prognostic factors to identify patients at higher risk of home epinephrine-treated reactions during milk OIT [92]. There are precedents for protocols allowing up-dosing at home which were shown to be safe [50, 93]. These usually involve more progressive up-dosing, with smaller but more frequent steps. However, it is important to note that studies on home-based OIT protocols were performed in carefully selected patients with high thresholds to peanut [50, 93].

Before considering up-dosing at home, it is essential to ensure that the parents have good OIT literacy and understand the risk associated with their decision [89]. Anaphylaxis action plan should be reviewed, individual risk factors such as asthma control should be addressed, and informed consent should be clearly documented in the patient’s chart. If the decision is made to proceed to up-dosing at home, it may be reasonable to consider more progressive up-dosing as to minimize risk [50, 93].

## Conclusions

The recent CSACI guidelines have emphasized the need that OIT be developed and practiced following the principle of patient-centered care. This goal can be achieved through the including shared decision-making approaches and the possibility to customize treatment in the general approach to OIT. The cases in this review show how the shared decision-making is the key to navigate the unavoidable areas of uncertainties in the management of OIT. While the practice of OIT is bound to evolve as additional scientific and experiential knowledge is gained, it should always remain rooted in the general principle of patient-centered care.

## Data Availability

Not applicable.

## Abbreviations

PCC: Patient-centered care
SDM: Shared decision-making
SPT: Skin-prick test
SSIgE: Serum-specific Immunoglobulin E
OFC: Oral food challenge
